# Smartphone addiction affects life satisfaction among Chinese university students: the serial mediation effects of social anxiety for social media users and mental well-being

**DOI:** 10.1186/s40359-025-03544-9

**Published:** 2025-10-30

**Authors:** Ting Jing, Soon-Yew Ju, Mohd Rozaimy Ridzuan, Lai-Kuan Kong, Noor Amira Syazwani Abd Rahman, Jing Li, Jianbin Xu, Mingxing Zhou

**Affiliations:** 1 Postdoctoral Research Station in Education, Southwest University, Chongqing, 400715 China; 2 Guangdong Preschool Normal College in Maoming, Maoming, 525000 Guangdong China; 3 Chongqing Yudongnan Academy of Agricultural Sciences, Chongqing, 408004 China; 4 Faculty of Administrative Science and Policy Studies, Universiti Teknologi MARA (UiTM) Pahang Branch, Raub Campus, Raub, Pahang 27600 Malaysia; 5 Faculty of Business and Management, Universiti Teknologi MARA (UiTM) Pahang Branch, Raub Campus, Raub, Pahang 27600 Malaysia; 6 School of Teacher Education, Heze University, Heze, 274000 China; 7 Chongqing College of International Business and Economics, Chongqing, 401520 China

**Keywords:** Life satisfaction, Mental Well-Being, Smartphone addiction, Social anxiety for social media users, University students

## Abstract

**Background:**

Life satisfaction is an essential component that enables individuals to discover meaning in their lives and attain cognitive well-being. Life satisfaction is typically associated with aspects such as meaningfulness, fulfilment, and happiness. To gain a deeper understanding of university students’ life satisfaction, this study explores the influence of psychological mechanisms of social anxiety for social media users and mental well-being from the perspective of smartphone addiction. Specifically, it aims to examine the effect of smartphone addiction on life satisfaction among university students in China, with social anxiety for social media users and mental well-being acting as serial mediators within the proposed research model.

**Methods:**

The respondent in this study was analyzed using a non-probability cross-sectional survey approach. Questionnaires were distributed to university students in China, which include the regions of Northeast China, North China, Northwest China, Central China, South China, East China, and Southwest China. Eventually, 4,159 university students were identified as usable data for analysis. The data were analyzed using IBM SPSS 29 and SmartPLS 4.1.1.4 software, specifically adopting the partial least squares-structural equation modelling (PLS-SEM) approach.

**Results:**

The results indicated that, for the direct effect hypotheses, smartphone addiction was not significantly associated with life satisfaction. However, smartphone addiction was positively and significantly associated with social anxiety related to social media use, and negatively and significantly associated with mental well-being. Social anxiety for social media users was negatively and significantly associated with both mental well-being and life satisfaction. Mental well-being, in turn, was positively and significantly associated with life satisfaction. Regarding the mediation and serial mediation hypotheses, the results showed that social anxiety for social media users and mental well-being both had significant negative indirect associations with the relationship between smartphone addiction and life satisfaction. Additionally, the serial mediation pathway through social anxiety and mental well-being was also statistically significant.

**Conclusions:**

This study contributes to a better understanding of the associations among smartphone addiction, social anxiety, mental well-being, and life satisfaction among university students. While causal interpretations cannot be drawn due to the cross-sectional design, the findings highlight potential psychological pathways that may underlie the link between intensive smartphone use and reduced life satisfaction. These insights may inform the development of future university-based interventions that aim to address smartphone-related difficulties and promote student well-being. As digital engagement continues to grow, further longitudinal research is recommended to clarify the directionality of these associations and to support the design of effective strategies for enhancing university students’ quality of life.

**Supplementary Information:**

The online version contains supplementary material available at 10.1186/s40359-025-03544-9.

## Introduction

Life satisfaction serves as a significant indicator of university students’ fulfilment in both academic and personal domains [1]. Promoting life satisfaction among university students has become a pressing and shared priority for governments, society, and academia. This issue is intricately linked to the physical and mental health of students at a pivotal developmental phase, with profound consequences for their future quality of life and the overall well-being of society [2]. Life satisfaction is a fundamental component essential for individuals to derive meaning from their existence and achieve cognitive well-being [3], and it is a primary characteristic of people’s living conditions [4]. Life satisfaction denotes an individual’s overall evaluation of their quality of life and psychological well-being [5]. The right to live a life where one is happy and fulfilled is a basic human right that should apply to everyone, regardless of their profession. Everyone, regardless of their social or economic status, studies, works, or is self-employed, wants to live a life that matters and brings them happiness. The life satisfaction of university students has become a significant indicator of student health, posing an enormous challenge for educational institutions to quantify due to its subjective nature [6].

The Sustainable Development Goals (SDGs) of the United Nations, especially those that deal with health, education, and well-being, are very similar to the idea of life satisfaction among university students. SDG 3: Good Health and Well-being, in particular, calls for everyone to have good mental health, human well-being, and caring for social equity [7]. University students who exhibit life satisfaction are more inclined to demonstrate academic motivation [8] and encounter less academic-related stress [9].

Although life satisfaction is essential for overall well-being and corresponds with global development objectives, the rising prevalence of smartphone usage, especially among university students, has elicited apprehensions regarding its possible effects on mental health and life satisfaction. Due to the swift advancement of the Internet, smartphones have become essential instruments for communication in daily life, particularly among the younger generation [5]. Individuals derive advantages from smartphone usage through its extensive array of applications that fulfil several roles, many of which might directly influence a person’s well-being and life satisfaction [10].

However, recent research indicates that smartphone addiction positively influences individual anxiety [11] and negatively affects life satisfaction [12]. Globally, the mental well-being of university students has deteriorated [13], and studies have indicated a strong correlation between students’ mental well-being and overall life satisfaction [14]. Considering the deteriorating mental health of university students worldwide and its significant correlation with life satisfaction, it is imperative to examine how behaviors such as smartphone addiction may exacerbate these difficulties, potentially worsening mental health conditions and reducing life satisfaction.

In China, the demographic of young individuals, particularly university students, is the most rapidly expanding segment of smartphone users [15]. University students frequently utilize smartphones for academic and daily tasks [16]. A 2018 survey revealed that Chinese university students allocate more than 5 h daily to mobile phone usage, with around 79% utilizing smartphones during class [17]. A survey of undergraduate students in China indicated a notable link between prolonged smartphone usage and diminished life satisfaction [12]. The above discussion suggests that excessive smartphone usage among university students may adversely affect their overall life satisfaction, likely due to disruptions in academic performance, social relationships, and mental well-being.

Existing research has primarily focused on the direct association between smartphone addiction and life satisfaction [18, 19], offering limited insight into the indirect pathways through which this effect may occur. While social anxiety has been recognized as a potential consequence of excessive smartphone use [20, 21], its mediating role in the link between smartphone addiction and life satisfaction has not been sufficiently explored. Similarly, although mental well-being is frequently acknowledged as a critical determinant of life satisfaction, its function as a mediator and particularly as part of a serial mediation pathway alongside social anxiety remains under-investigated [3]. This study adopts a serial mediation model that differs from prior research. For example, Samaha and Hawi [18] used perceived stress and academic performance as mediators but lacked a theoretical basis. Su and He [22] employed a simple mediation with loneliness, while Zhu et al. [23] used parallel mediation involving negative emotions. Ding et al. [24] and Chen et al. [25] focused on correlational and moderation effects, respectively, without exploring mediation pathways. In contrast, the present study advances theoretical understanding by applying the Stress Process Model, linking smartphone addiction to life satisfaction through social anxiety and mental well-being in a sequential process, offering a deeper insight into the psychological mechanisms at play.

Moreover, much of the current literature lacks a comprehensive theoretical framework to explain how stress-related outcomes such as anxiety and poor mental well-being evolve from digital overuse and impact broader life outcomes like life satisfaction. This study adopts the Stress Process Model (SPM) [26] as the foundational theoretical framework to examine how smartphone addiction influences psychological outcomes among Chinese university students. The SPM conceptualizes stress as a dynamic and multidimensional process, consisting of three major domains, namely sources of stress, mediators of stress, and manifestations of stress [26]. Within the domain of stress sources, the present study identifies smartphone addiction as a modern, chronic behavioral stressor. The mediators of stress in the SPM include both secondary stressors and psychological resources that influence how individuals respond to initial stress exposure [26, 27]. In this model, social anxiety is conceptualized as a secondary stressor that may arise from smartphone overuse.

Simultaneously, mental well-being is considered a key psychological resource, a buffer that typically helps individuals cope with stress. However, prolonged exposure to behavioral stressors (smartphone addiction) and social anxiety can erode this resource, reducing an individual’s capacity to maintain emotional stability and positive functioning. Finally, the manifestation of stress is captured through life satisfaction, a global measure of subjective well-being.

By structuring the research model as a serial mediation pathway from smartphone addiction to social anxiety, to mental well-being, and ultimately to life satisfaction, this study extends the SPM into the context of digital behavior and university student mental health. This approach not only highlights the complex psychological mechanisms linking smartphone addiction to broader life outcomes but also responds to calls for more theory-driven research on the mental health implications of technology use in non-Western populations. The findings of the study will guide academic and policy measures aimed at enhancing mental health and well-being among students, following the United Nations’ Sustainable Development Goal 3: Good Health and Well-being, which emphasizes the mental health of youth. This research ultimately enhances global initiatives aimed at improving university students’ mental health and life satisfaction, which are crucial for cultivating a more resilient, engaged, and productive future workforce.

## Literature review

### Smartphone addiction and life satisfaction

The phrase “smartphone addiction” is a problem wherein individuals exhibit excessive, compulsive, or detrimental behaviors linked to their smartphone usage [28, 29]. Smartphone addiction often leads to emotional exhaustion, reduced face-to-face social interactions, and constant comparison through social media, all of which can diminish one’s sense of fulfillment and contentment with life [29, 30]. Samaha and Hawi [18] found that smartphone addiction is positively associated with perceived stress, which in turn negatively affects life satisfaction. Mathurawala [19] carried out a study among undergraduate students in Pune, India, involving 88 participants, and revealed a significant negative correlation between smartphone addiction and life satisfaction. Jiang et al. [12] conducted a study among Chinese university students and found a negative association between excessive mobile phone use and life satisfaction. Therefore, based on both empirical findings and theoretical reasoning, the following proposition can be made:

- H1: Smartphone addiction negatively influences life satisfaction.

### Smartphone addiction and social anxiety for social media users

Social anxiety, often known as social phobia, is a disorder defined by an intense dread of being observed or embarrassed by others [31]. Smartphone addiction may exacerbate social anxiety, particularly among individuals who utilize social media. Thatkar et al. [21] did a study including 464 young college students and identified a favorable association between smartphone addiction and social anxiety. Mohd Salleh Sahimi [20] similarly emphasized that social anxiety impedes face-to-face contact, and their findings indicated a substantial correlation between excessive smartphone use and an elevated risk of social anxiety. The relationship between social media addiction and social anxiety is complex, with mixed findings on its directionality. Some studies suggest addiction worsens social anxiety through increased fear of negative evaluation [32], while others propose that social anxiety may lead to greater addiction as individuals use social media to avoid real-life interactions [33]. This study treats smartphone addiction as a stress source that increases social anxiety, consistent with the Stress Process Model. Based on the empirical facts and theoretical frameworks, it is fair to assert that:

- H2: Smartphone addiction positively influences social anxiety for social media users.

### Smartphone addiction and mental well-being

Abuhamdah and Naser [34] conducted a study on university students in Jordan, revealing that excessive smartphone usage severely affects both physical and mental health. Excessive smartphone usage and addiction to these devices have been demonstrated to adversely impact people’s physical and mental health. Susmitha et al. [35] discovered that smartphone addicts exhibited inferior mental well-being relative to non-addicts. Consequently, smartphone addiction can adversely impact mental health by elevating stress levels, disturbing sleep patterns, and impairing emotional regulation. Excessive reliance on smartphones may diminish psychological resilience and disrupt good habits and relationships, essential for sustaining mental health. Considering these psychological and behavioral ramifications, it is logical to deduce that:

- H3: Smartphone addiction negatively influences mental well-being.

### Social anxiety for social media users and mental well-being

Anxiety is among the most prevalent mental health issues in contemporary society [36]. Russell and Topham [37] contended that social anxiety is a widespread mental health concern that exists along a continuum of suffering and impairment. Social media can intensify social anxiety by promoting excessive self-scrutiny and strengthening social apprehensions [38]. This heightened anxiety frequently results in adverse mental health consequences, such as sadness, isolation, and diminished life satisfaction [39]. Consequently, it can be concluded that increased struggles with social anxiety correlate with a decline in mental well-being. Consequently, it is hypothesized that:

- H4: Social anxiety for social media users negatively influences mental well-being.

### Social anxiety for social media users and life satisfaction

Life satisfaction is seen as a fundamental element of psychological well-being. Research indicates that elevated anxiety correlates with diminished life satisfaction [40]. Collins [41] conducted research at the University of Essex, revealing a negative link between social anxiety and life satisfaction among social media users. In other words, the elevated social anxiety correlated with diminished life satisfaction. Chin et al. [42] found that individuals with social anxiety disorder experienced significantly reduced positive affect—including joy, contentment, and amusement, highlighting a diminished capacity for enjoyment even in typically pleasurable contexts.This indicates that heightened social anxiety correlates with diminished life satisfaction. In light of this discourse and prior empirical findings, the subsequent hypothesis is posited:

- H5: Social anxiety for social media users negatively influences life satisfaction.

### Mental well-being and life satisfaction

Mental well-being is a condition of health when an individual fully realizes their potential, effectively manages daily life challenges, and contributes positively to the advancement of their community [43]. Previous research [44, 45] identified a strong inverse correlation between life satisfaction and the existence of mental illness. Conversely, additional research [46, 47] indicated that mental well-being had a favorable impact on persons’ life satisfaction. Lombardo et al. [48] conducted a study including 400,000 Canadians and discovered a strong correlation between self-reported mental health and life satisfaction, even after controlling for variables such as income, general health, and gender. It was discovered that those with poor mental health exhibited notably low life satisfaction.

Nagar and Saxena [47] conducted a study with 200 young individuals in India, revealing that mental well-being positively affects life satisfaction. The preceding explanation suggests that mental well-being facilitates the experience of happy emotions, fosters robust connections, and sustains a sense of purpose in life, all of which are essential elements of life pleasure. Moreover, when mental well-being is elevated, individuals are more adept at managing stress and adapting to life’s obstacles, therefore improving their overall life pleasure. Based on the presented reasons, it is asserted that:

- H6: Mental well-being positively influences life satisfaction.

### Smartphone addiction, social anxiety for social media users, and life satisfaction

Smartphone addiction undeniably detrimentally affects life satisfaction [18, 19]. Previous research [20, 21] has demonstrated that smartphone addiction positively affects social anxiety in social media users. Conversely, social anxiety in social media users was discovered to adversely affect life satisfaction [40, 41]. Current literature indicates that smartphone addiction directly diminishes life satisfaction. Simultaneously, it exacerbates social anxiety among social media users, which has been demonstrated to adversely impact life satisfaction. The findings suggest a possible indirect mechanism by which smartphone addiction affects life satisfaction through social anxiety. Consequently, investigating the mediating function of social anxiety may yield a more thorough comprehension of the psychological ramifications of smartphone addiction and furnish profound insights into its impact on users’ overall life satisfaction. Therefore, it is logical to suggest that:

- H7: Social anxiety for social media users mediates the relationship between smartphone addiction and life satisfaction.

### Smartphone addiction, mental well-being and life satisfaction

Previous research indicates that smartphone addiction adversely affects life satisfaction [18, 19]. Smartphone addiction has been shown to adversely impact mental well-being [28, 29, 34, 35]. Furthermore, previous research [46–48] has demonstrated that mental well-being favorably affects individuals’ life satisfaction. The findings suggest that mental well-being may function as a psychological mechanism that ties the relationship between smartphone addiction and life satisfaction.Therefore, it is hypothesized that:

- H8: Mental well-being mediates the relationship between smartphone addiction and life satisfaction.

### Smartphone addiction, social anxiety for social media users, mental well-being and life satisfaction

Life satisfaction is the comprehensive assessment of a person’s well-being and quality of life, and numerous psychological and behavioral aspects can influence this outcome through various interrelated pathways. Smartphone addiction, defined by compulsive and excessive smartphone use, has been associated with increased social anxiety [49]. This increased social anxiety can harm a person’s mental health and lead to reduced emotional stability and resilience [50]. Consequently, diminished mental well-being has been consistently associated with lower life satisfaction [47]. Furthermore, social anxiety and mental well-being may sequentially mediate the relationship between smartphone addiction and life satisfaction. Based on the above, it is hypothesized that social anxiety and mental well-being act as serial mediators in the relationship between smartphone addiction and life satisfaction. This leads to the hypothesis that:

- H9: The relationship between smartphone addiction and life satisfaction is serially mediated by social anxiety for social media users and mental well-being.

**Fig. 1:**
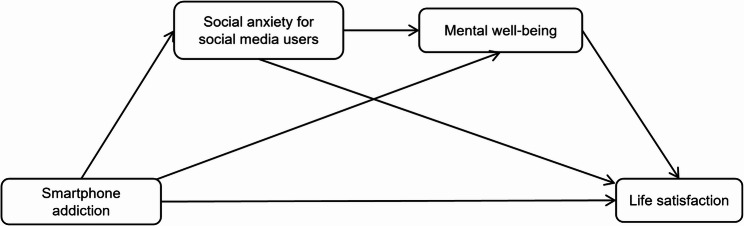
Hypothetical model

Based on the literature review and the above discussion, the serial mediation model is presented in Fig. 1.

## Research methods

### Instrumentation

#### Smartphone addiction scale – short version (SAS-SV)

This study adopted the Chinese version of the Smartphone Addiction Scale – Short Version (SAS-SV) as the measurement tool [51]. The scale has been widely used to assess individuals’ levels of smartphone addiction in daily life and demonstrates strong cross-cultural applicability [52]. The SAS-SV is a unidimensional scale consisting of 10 items, each rated on a 5-point Likert scale (1 = “strongly disagree” to 5 = “strongly agree”). Higher scores indicate a greater tendency toward smartphone addiction. The Cronbach’s alpha coefficient in this study was 0.937, indicating a high level of internal consistency and demonstrating that the scale is a reliable instrument for capturing addictive smartphone use behaviors among university students.

#### Social anxiety for social media users (SAS-SMU)

The Social Anxiety Scale for Social Media Users (SAS-SMU) is specifically designed to assess the level of social anxiety individuals experience while using social media platforms. The scale has been translated into Chinese and validated for use among Chinese university students [53]. The Chinese version consists of 21 items that cover four key dimensions of social anxiety related to social media use: Sharing Content Anxiety (SCA, 7 items), Privacy Concern Anxiety (PCA, 5 items), Interaction Anxiety (IA, 6 items), and Self-Evaluation Anxiety (SEA, 3 items). Each item is rated on a 7-point Likert scale (1 = “strongly disagree,” 7 = “strongly agree”), with higher scores indicating greater levels of social anxiety. The scale demonstrates excellent internal consistency, with Cronbach’s alpha values from 0.925 to 0.957, indicating that it is a reliable instrument for comprehensively measuring different types of social anxiety associated with social media use.

#### Warwick-Edinburgh mental Well-being scale (WEMWBS)

The Warwick-Edinburgh Mental Well-being Scale (WEMWBS) is a commonly used instrument for assessing individuals’ levels of positive mental well-being and has demonstrated good reliability and validity [54]. The scale adopts a unidimensional structure and consists of 14 positively worded items, each rated on a 7-point Likert scale. Higher scores indicate higher levels of positive mental well-being. In the present study, the Cronbach’s alpha coefficient was 0.978.

#### Satisfaction with life scale (SWLS)

The Satisfaction with Life Scale (SWLS) was extensively utilised to assess life satisfaction [55]. It comprises a total of five items, such as “In most ways my life is close to my ideal”. A prior study with college students in Western China indicated that the Cronbach’s alpha coefficient for this scale was 0.920 [56]. The seven-point Likert scale was utilised to evaluate the items, with 1 indicating complete disagree and 7 indicating completely agree. The Cronbach’s alpha in the present study was 0.925.

#### Sampling and data collection

This study applied the non-probability purposive sampling method and focused on undergraduate university students in China. Data were gathered from Northeast China, North China, Northwest China, Central China, South China, East China, and Southwest China. The researcher published the questionnaire on the online platform and distributed the generated link or QR code to the targeted respondents. Instructions were included in the questionnaire, and participants were informed that their participation in the study was voluntary, allowing them to withdraw at any point throughout the completion of the questionnaire. Respondents who completed the questionnaire were deemed to have implicitly consented to participate in this study.

According to Gefen [57], the sample size must be adequate to provide the required statistical power. The G*Power software may assist the researcher in determining the minimum sample size needed prior to data collection [58]. To estimate the sample size needed for this study, a priori power analysis [59, 60] embedded in G*Power software is used for that purpose. Based on the most conservative scenario of input parameters of a medium effect size (*f*^2^), with an α of 0.05, power of 0.95 for this serial mediation model, the optimum sample size needed for the study model is 776 respondents. Hence, this study collected 4,159 completed usable questionnaires, therefore eliminating concerns regarding sample size.

Initially, data were successfully collected from 4,650 respondents. However, prior to analysis, the dataset was screened for questionable response patterns. Specifically, responses displaying odd or mechanical answering behaviors, such as straight ascending, straight descending, or straight-lining. These cases were excluded to ensure data quality. After this screening process, 4,159 valid responses (89.44%) from university students in China remained for analysis.

Among the 4,159 valid respondents, 37% (*n* = 1,537) were male and 63% (*n* = 2,622) were female. In terms of academic pathway, 59% (*n* = 2,452) were enrolled in degree programs, while 41% (*n* = 1,707) were pursuing vocational education. A large majority of the respondents (99%; *n* = 4,119) were aged 25 years and below, which is logical and justifiable given that most undergraduate students fall within this age range; only 1% (*n* = 40) were aged above 25. Regarding locality, 68.3% (*n* = 2,841) of the students were from rural areas, whereas 31.7% (*n* = 1,318) were from urban areas. Additionally, 74.8% (*n* = 3,113) of the students reported having siblings, while 25.2% (*n* = 1,046) were from single-child families. Regarding regional distribution, the majority of respondents were from South China, comprising 27% (*n* = 1,123) of the sample. This was followed by North China with 25% (*n* = 1,041), Central China with 23% (*n* = 955), East China with 10.2% (*n* = 426), Southwest China with 7.8% (*n* = 326), Northwest China with 5.3% (*n* = 222), and Northeast China with the smallest proportion at 1.6% (*n* = 66).

#### Control variables

To enhance the robustness of the structural model and minimize potential bias, several control variables that may influence life satisfaction were incorporated into the data analysis. In particular, demographic factors have been widely acknowledged in prior research as important determinants of life satisfaction. Accordingly, this study controlled for gender [61, 62], age [63], education level [64], and locality of urban-rural residence [65, 66]. In addition, family-related characteristics, such as single-child versus multiple-child background [67], and regional differences [68] were also included. By accounting for these demographic variables, the analysis ensures that the observed effects on life satisfaction are not confounded by background characteristics, thereby providing a more reliable assessment of the hypothesized relationships. Controlling for these variables helps account for potential confounding effects, allowing for a clearer examination of the unique impacts of the primary variables under investigation and enhancing the robustness and validity of the study’s findings. Statistically partialing out several confounders can help even though it would not resolve the problem completely [69]. By controlling for these demographic variables, the present study ensures that the estimated effects on life satisfaction more accurately reflect the hypothesized relationships.

#### Data analysis

This study utilized two primary statistical software packages for data analysis: SPSS 29 and SmartPLS 4.1.1.4 [70]. The latter was employed to conduct variance-based structural equation modeling (VBSEM) using partial least squares structural equation modeling (PLS-SEM). VB-SEM was utilised as the primary aim of this study is to estimate key target variables and establish an explanatory model. Additionally, the study seeks to identify component scores that account for as much variance as possible in the endogenous constructs. Furthermore, this research model is relatively complex, comprising 50 indicators across 7 constructs and incorporating a higher-order reflective structure. For these reasons, the researcher determined that the PLS-SEM approach is most appropriate [71].

## Results

### Common method bias

For this study, precautions were taken to address the issue of common method bias (CMB) prior to conducting the assessment of the measurement model and the structural model, considering that the same respondents answered all items for both exogenous and endogenous variables. Two methods were employed to detect potential common method variance. Firstly, a procedural remedy was implemented by using different Likert scales for the exogenous and endogenous variables [72]. Secondly, a statistical remedy was applied through the full collinearity assessment approach as recommended by Kock [73]. The results of the full collinearity test, as presented in Table 1, indicate that all variance inflation factor (VIF) values for both exogenous and endogenous variables were below the threshold of 5 [74]. The VIF values ranged from 1.400 for SAS to 3.869 for SASSMUSEA, suggesting that CMB was not a significant threat in this study.

**Table 1:** Full covariance test

SAS	SASSMUIA	SASSMUPCA	SASSMUSCA	SASSMUSEA	MWB	SWLS
1.400	3.330	2.200	3.524	3.869	1.853	1.723

### Measurement model assessment

Once it was confirmed that all latent variables had been tested without any issues of common method bias, as evidenced in Table 1, the measurement model was evaluated by examining all fifty indicator loadings for the first-order dimensions and the four indicator loadings for the higher-order dimension, as presented in Table 2. Hair et al. [75] recommended that indicator loadings exceed 0.708, although loadings above 0.50 may still be deemed acceptable. In addition, construct reliability was assessed by examining Cronbach’s alpha (CA) and composite reliability (CR), ensuring that both metrics surpassed the 0.70 threshold [76]. Following these procedures, both convergent and discriminant validity were evaluated. For convergent validity, the average variance extracted (AVE) was evaluated, and it was ensured that each AVE value exceeded the 0.50 threshold [75]. Finally, discriminant validity was assessed using the heterotrait–monotrait ratio (HTMT) method, as recommended by Henseler et al. [77]. According to Henseler et al. [77], discriminant validity is established when the HTMT value is at or below 0.90, in line with the data analysis procedures adopted by Li et al. [78]and Ridzuan et al. [79].

**Fig. 2:**
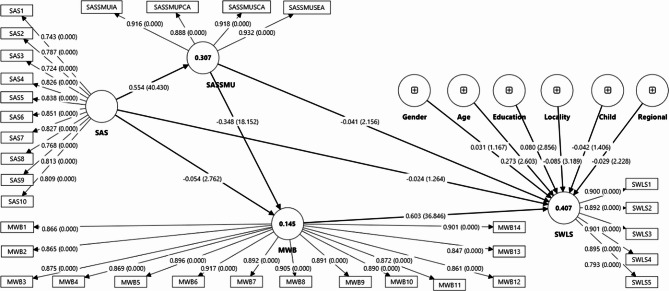
Hypothesis testing

The results presented in Table 2 reveal that the factor loadings of the first-order indicators range from 0.724 (for SAS3) to 0.939 (for SASSMUSEA20). In contrast, the loadings for the higher-order dimension span from 0.888 (for SASSMUPCA) to 0.932 (for SASSMUSEA). The indicator loadings reported in Table 2 demonstrate that all fifty first-order indicators and all four higher-order indicators exceeded the recommended cutoff of 0.708. All constructs demonstrated strong convergent validity, with AVE values exceeding the 0.50 benchmark and ranging from 0.639 for SAS to 0.870 for SASSMUSEA. The graphical representation of each indicator’s loading is illustrated in Fig. 2.

Reliability was likewise robust, in which both CA and CR values were assessed. Table 2 presents the CA and CR values for each construct, in which SAS (0.937, 0.946), SASSMUIA (0.957, 0.965), SASSMUPCA (0.949, 0.961), SASSMUSCA (0.952, 0.961), SASSMUSEA (0.925, 0.952), MWB (0.978, 0.980), SWLS (0.925, 0.943) and SASSMU (0.934, 0.953). All of the lower-order and higher-order constructs have exceeded the criterion of 0.70 [76, 80]. Taken together, the results in Table 2 confirm that the measurement model meets the criteria for indicator loadings, internal consistency reliability, and convergent validity for lower-order dimensions and higher-order dimensions.

**Table 2:** Construct reliability and convergent validity assessment

Construct	Indicators	Loading	CA	CR	AVE
Smartphone addiction (SAS)	SAS1	0.743	0.937	0.946	0.639
	SAS2	0.787			
	SAS3	0.724			
	SAS4	0.826			
	SAS5	0.838			
	SAS6	0.851			
	SAS7	0.827			
	SAS8	0.768			
	SAS9	0.813			
	SAS10	0.809			
Social anxiety for social media users	SASSMUIA1	0.912	0.957	0.965	0.822
interaction anxiety (SASSMUIA)	SASSMUIA2	0.913			
	SASSMUIA3	0.927			
	SASSMUIA4	0.893			
	SASSMUIA5	0.889			
	SASSMUIA6	0.906			
Social anxiety for social media users	SASSMUPCA7	0.919	0.949	0.961	0.830
privacy concern anxiety (SASSMUPCA)	SASSMUPCA8	0.927			
	SASSMUPCA9	0.916			
	SASSMUPCA10	0.889			
	SASSMUPCA11	0.904			
Social anxiety for social media users	SASSMUSCA12	0.824	0.952	0.961	0.777
sharing content anxiety (SASSMUSCA)	SASSMUSCA13	0.898			
	SASSMUSCA14	0.915			
	SASSMUSCA15	0.893			
	SASSMUSCA16	0.829			
	SASSMUSCA17	0.912			
	SASSMUSCA18	0.896			
Social anxiety for social media users self-	SASSMUSEA19	0.922	0.925	0.952	0.870
evaluation anxiety (SASSMUSEA)	SASSMUSEA20	0.939			
	SASSMUSEA21	0.937			
Mental well-being (MWB)	MWB1	0.866	0.978	0.980	0.778
	MWB2	0.865			
	MWB3	0.875			
	MWB4	0.869			
	MWB5	0.896			
	MWB6	0.917			
	MWB7	0.892			
	MWB8	0.905			
	MWB9	0.891			
	MWB10	0.890			
	MWB11	0.872			
	MWB12	0.861			
	MWB13	0.847			
	MWB14	0.901			
Life satisfaction (SWLS)	SWLS1	0.900	0.925	0.943	0.769
	SWLS2	0.892			
	SWLS3	0.901			
	SWLS4	0.895			
	SWLS5	0.793			
Social anxiety for social media users	SASSMUIA	0.916	0.934	0.953	0.834
(SASSMU) – Higher-Order	SASSMUPCA	0.888			
	SASSMUSCA	0.918			
	SASSMUSEA	0.932			

Following the measurement model assessment, discriminant validity was validated using the heterotrait-monotrait ratio (HTMT), as recommended by Henseler et al. [77], and this approach was also employed in the study by Li et al. [81]. The results reported in Table 3 indicate that all HTMT values fall below 0.90. Consequently, discriminant validity in this study’s model is verified and established, confirming that the model does not violate the discriminant validity threshold.

**Table 3:** Discriminant validity assessment (HTMT)

Constructs	SAS	SASSMUIA	SASSMUPCA	SASSMUSCA	SASSMUSEA	MWB
SASSMUIA	0.536					
SASSMUPCA	0.521	0.767				
SASSMUSCA	0.530	0.806	0.824			
SASSMUSEA	0.561	0.887	0.795	0.861		
MWB	0.257	0.377	0.308	0.329	0.412	
SWLS	0.208	0.261	0.281	0.253	0.307	0.654

### Structural model

After establishing the absence of common method bias and confirming that the measurement model met the required thresholds for indicator loadings, reliability, convergent validity, and discriminant validity, this study proceeded to test the structural model. This two-step approach, as outlined by Anderson [82] and following the data analysis guidelines conducted by Li et al. [83], Ridzuan et al. [84], and Ju et al. [85], first involves validating the measurement model’s reliability and validity before evaluating the structural model. The structural model assessment focused on the magnitude of the hypothesized path coefficients for both direct and indirect effects, employing a bootstrapping procedure with 10,000 resamples in accordance with Guenther et al. [86]. Collinearity within the structural model was inspected via VIF values, with a recommended stringent cutoff of less than 3.3 to confirm the absence of multicollinearity among predictor variables [87]. Table 4 indicates that all VIF values are below 3.3.

In addition, this study assesses the explanatory power of the model by looking at the R^2^ value and the study also evaluated effect sizes (f²) to determine the influence of each exogenous variable on the endogenous constructs, applying Cohen’s [88] benchmarks for small (0.02), medium (0.15), and large (0.35) effects. Next, the model’s predictive relevance was assessed by examining Q² values, and its predictive power was evaluated using the PLS-Predict procedure [89].

To evaluate the potential influence of demographic characteristics, six control variables were incorporated into the structural model, i.e., gender, age, education level, locality of urban-rural residence, single-child versus multiple-child background, and regional differences. The analysis revealed that four of these variables were statistically significant predictors of life satisfaction, namely age (β = 0.273, t = 2.603, *p* = 0.005), education level (β = 0.080, t = 2.856, *p* = 0.002), urban-rural residence (β=−0.085, t = 3.189, *p* = 0.001), and regional differences (β=−0.029, t = 2.228, *p* = 0.013), while gender (β = 0.031, t = 1.167, *p* = 0.122) and single-child versus multiple-child background (β=−0.042, t = 1.406, *p* = 0.080) had no significant impact on life satisfaction. Importantly, the inclusion of these six control variables did not substantially alter the strength and the significance of the hypothesized path relationships among the focal constructs of this study. This indicates that the proposed model is robust, hence, the subsequent analysis focuses on the structural model results for the primary constructs of interest.

Tables 4 and 5 present the direct and indirect effects of the study’s hypotheses, respectively. Six direct-effect hypotheses were evaluated in Table 4, and three indirect-effect hypotheses were tested in Table 5. (H1) Smartphone addiction has no significant effect on life satisfaction (β=−0.024, t = 1.264, LL=−0.054, UL = 0.007, *p* = 0.103). (H2) Smartphone addiction has a positive and significant effect on social anxiety for social media users (β = 0.554, t = 40.430, LL = 0.531, UL = 0.576, *p* = 0.000). (H3) Smartphone addiction has a negative and significant effect on mental well-being (β=−0.054, t = 2.762, LL=−0.086, UL=−0.022, *p* = 0.003). (H4) Social anxiety for social media users has a negative and significant effect on mental well-being (β=−0.348, t = 18.152, LL=−0.379, UL=−0.316, *p* = 0.000). (H5) Social anxiety for social media users has a negative and significant effect on life satisfaction (β=−0.041, t = 2.156, LL=−0.074, UL=−0.011, *p* = 0.016). (H6) Mental well-being has a positive and significant effect on life satisfaction (β = 0.603, t = 36.846, LL = 0.575, UL = 0.629, *p* = 0.000). Furthermore, for the direct hypotheses H2 to H6, neither the lower limit (LL) nor the upper limit (UL) includes zero. Hence, these results suggested that the direct hypotheses of H2 to H6 were supported. The presentation is shown graphically in Fig. 2.

To provide a clearer understanding of the research framework, this study developed two indirect-effect hypotheses and one serial mediation hypothesis, in which social anxiety for social media users and mental well-being serve as mediators and serial mediators, respectively, in the relationship between smartphone addiction and university students’ life satisfaction. The study’s findings are presented in Table 5. The results showed that (H7) the indirect effect of social anxiety for social media users on the link between smartphone addiction and life satisfaction was negative and significant (β=−0.023, t = 2.162, LL=−0.041, UL=−0.006, *p* = 0.015). (H8) The indirect effect of mental well-being on the link between smartphone addiction and life satisfaction was negative and significant (β=−0.033, t = 2.785, LL=−0.052, UL=−0.013, *p* = 0.003). (H9) The third serial mediation effect that passes through social anxiety for social media users and mental well-being serially, with the former influencing the latter, reaches significance (β= −0.116, t = 14.439, LL=−0.130, UL=−0.104, *p* = 0.000). Furthermore, for all three indirect hypotheses, neither the lower limit (LL) nor the upper limit (UL) includes zero. Hence, these results supported all of the indirect-effect hypotheses and serial mediation hypotheses from H7 to H9.

The explanatory power of the model was assessed. The results revealed that 40.7% of the variance of life satisfaction was explained by smartphone addiction, social anxiety for social media users and mental well-being, 14.5% of the variance of mental well-being was explained by smartphone addiction and social anxiety for social media users, and lastly 30.7% of the variance of social anxiety for social media users was explained by smartphone addiction. Mental well-being has a large effect on life satisfaction (f^2^ = 0.516), and smartphone addiction also has a large effect on social anxiety for social media users (f^2^ = 0.444), since f^2^ is greater than 0.35. Social anxiety for social media users has a small effect on mental well-being (f^2^ = 0.098) and life satisfaction (f^2^ = 0.02), and lastly smartphone addiction has a small effect on mental well-being (f^2^ = 0.02) as the value of f^2^ is greater than 0.02 and less than 0.15 [88].

**Table 4:** Direct effect analysis

Hypothesis	Relationship	Beta	SE	T values	*P* values	LL	UL	VIF	*R*²	F²	Decision
H1	SAS ->SWLS	−0.024	0.019	1.264	0.103	−0.054	0.007	1.451	0.407		Not Supported
H2	SAS ->SASSMU	0.554	0.014	40.430	0.000	0.531	0.576	1.000	0.307	0.444	Supported
H3	SAS ->MWB	−0.054	0.020	2.762	0.003	−0.086	−0.022	1.444	0.145	0.002	Supported
H4	SASSMU ->MWB	−0.348	0.019	18.152	0.000	−0.379	−0.316	1.444		0.098	Supported
H5	SASSMU ->SWLS	−0.041	0.019	2.156	0.016	−0.074	−0.011	1.594		0.002	Supported
H6	MWB ->SWLS	0.603	0.016	36.846	0.000	0.575	0.629	1.189		0.516	Supported

Note: *SAS* Smartphone addiction, *MWB* Mental well-being, *SWLS* Life satisfaction, *SASSMU* Social anxiety for social media users, Lower limit (*LL*), Upper limit (*UL*)

**Table 5:** Indirect effect analysis

Hypothesis	Relationship	Beta	SE	T values	*P* values	LL	UL	Decision
H7	SAS ->SASSMU ->SWLS	−0.023	0.011	2.162	0.015	−0.041	−0.006	Supported
H8	SAS ->MWB ->SWLS	−0.033	0.012	2.785	0.003	−0.052	−0.013	Supported
H9	SAS ->SASSMU ->MWB ->SWLS	−0.116	0.008	14.439	0.000	−0.130	−0.104	Supported

Note: *SAS* Smartphone addiction, *MWB* Mental well-being, *SWLS* Life satisfaction, *SASSMU* Social anxiety for social media users, Lower limit (*LL*), Upper limit (*UL*)

The PLS predict technique was employed to forecast measurement error by comparing the Root Mean Square Error (RMSE) of PLS with that of Linear Modelling (LM), as recommended by Shmueli et al. [89], in order to assess the predictive power of the model. Table 6 demonstrated a small predictive power in the model, since the majority of the items for all exogenous variables in the PLS predict analysis exceed the values derived from the LM model [75]. The data in Table 6 indicated that all Q² values were above 0, signifying adequate predictive relevance. The evaluation techniques were utilized in the study conducted by Jiang et al. [90].

**Table 6:** PLS predict

Indicators	Q²predict	PLS-SEM_RMSE	LM_RMSE	PLS-LM
MWB1	0.042	1.439	1.438	0.001
MWB2	0.042	1.452	1.453	−0.001
MWB3	0.048	1.499	1.497	0.002
MWB4	0.042	1.440	1.438	0.002
MWB5	0.054	1.483	1.483	0.000
MWB6	0.061	1.405	1.404	0.001
MWB7	0.060	1.415	1.415	0.000
MWB8	0.045	1.451	1.450	0.001
MWB9	0.048	1.489	1.488	0.001
MWB10	0.049	1.513	1.509	0.004
MWB11	0.045	1.352	1.352	0.000
MWB12	0.046	1.446	1.447	−0.001
MWB13	0.028	1.374	1.372	0.002
MWB14	0.045	1.422	1.422	0.000
SASSMUIA	0.258	0.858	0.853	0.005
SASSMUPCA	0.241	0.866	0.864	0.002
SASSMUSCA	0.250	0.861	0.860	0.001
SASSMUSEA	0.273	0.850	0.848	0.002
SWLS1	0.035	1.449	1.444	0.005
SWLS2	0.028	1.442	1.443	−0.001
SWLS3	0.037	1.410	1.411	−0.001
SWLS4	0.024	1.564	1.555	0.009
SWLS5	0.018	1.737	1.718	0.019

## Discussion

This study proposes a serial mediation effect model wherein smartphone addiction serves as the predictor, social anxiety for social media users and mental well-being function as serial mediators, and life satisfaction of university students is the outcome. This model was confirmed through statistical analysis of questionnaire data from Chinese university students. This study’s discussion section is segmented into three components: direct predictive effects, mediating effects, and serial mediating effects.

### Direct predictive effects

This study tested six direct effect hypotheses. Among them, the first hypothesis, linking smartphone addiction directly to life satisfaction, was not supported, while the remaining five were supported. The H1 was not supported, potentially due to the indirect influence of smartphone addiction on life satisfaction, mediated by psychological factors such as social anxiety and mental well-being. In Chinese universities, extensive smartphone usage is commonly accepted [16] and is not regarded as detrimental unless it results in psychological distress. This could explain the insignificant direct relationship with life satisfaction, despite a significant indirect effect through the mediators. Regarding H2, the findings indicate that smartphone addiction increases social anxiety among social media users. This supports prior research showing that problematic smartphone use intensifies social anxiety and fear of negative evaluation [20, 21]. Compulsive social media interaction fosters social comparison and perceived inadequacy, both of which contribute to social anxiety [91]. Supporting H3, the results also show that smartphone addiction negatively affects mental well-being. This aligns with previous findings linking excessive smartphone use to emotional fatigue, disrupted sleep, and increased stress [34, 91]. Such patterns often displace essential activities like sleep, physical exercise, and meaningful offline interactions, particularly important for the mental health of Chinese university students experiencing academic and social pressures.

The study corroborated H4, showing that social anxiety among social media users negatively impacts mental well-being. This finding aligns with theoretical perspectives suggesting that social anxiety can impair emotional functioning, lower self-esteem, and increase loneliness [38, 39]. Social media often exacerbates these issues by promoting self-scrutiny and reinforcing social fears, which can contribute to negative mental health outcomes such as sadness, isolation, and reduced life satisfaction. H5 was also supported, indicating that social anxiety in social media contexts negatively affects life satisfaction. This supports prior research suggesting that individuals with high levels of social anxiety may struggle to form meaningful connections online, leading to lower life satisfaction [92]. Finally, H6 was validated, demonstrating that mental well-being positively influences life satisfaction. This result is consistent with existing literature, which frequently highlights that individuals with higher mental well-being report greater life satisfaction [46–48].

### Mediating effects

This study’s results offer significant insights into the intricate links among smartphone addiction, social anxiety for social media users, mental well-being and life satisfaction in Chinese university students. The results support the proposed hypotheses (H7 and H8). Social anxiety for social media users negatively mediates the association between smartphone addiction and life satisfaction (H7). Compulsive social media usage frequently exacerbates social anxiety, particularly in China, where social comparison is widespread. This anxiety adversely impacts life satisfaction. Interventions should not solely concentrate on diminishing screen time but also assist students in managing their social media engagement and promoting offline social interactions. Mental well-being negatively mediates the association between smartphone addiction and life satisfaction (H8), suggesting that excessive smartphone usage detrimentally affects students’ emotional health. This aligns with evidence indicating that smartphone addiction results in adverse mental health effects. For Chinese university students, where academic and social success is paramount, smartphone addiction supplants healthy activities and undermines mental well-being, thereby lowering life happiness. Mitigating smartphone addiction in universities may enhance students’ mental well-being and overall quality of life.

### Serial mediating effect

This study investigates the serial mediation role of social anxiety for social media users and mental well-being in the link between smartphone addiction and life satisfaction (H9). The results highlight the substantial impact of social anxiety for social media users and mental well-being in elucidating the relationship between smartphone addiction and life satisfaction, especially among Chinese university students. This suggests that smartphone addiction adversely affects life satisfaction among Chinese university students via the serial mediating roles of social anxiety for social media users and mental well-being.

Smartphone addiction in Chinese university students, fueled by excessive engagement with social media platforms such as WeChat, QQ, and Weibo, exacerbates social anxiety through incessant social comparison and the compulsion to uphold a favorable online persona. This intensified social anxiety, driven by the apprehension of negative assessment and the quest for affirmation, exacerbates mental health concerns, resulting in emotional fatigue, isolation, and melancholy. The ensuing identity confusion between authentic and virtual personalities, alongside scholastic and social expectations, undermines their self-esteem and mental well-being. These mental health difficulties adversely impact life satisfaction.

Consistent with the Stress Process Model [26], the findings of the present study demonstrate that smartphone addiction acts as a primary stressor that indirectly affects life satisfaction through sequential mediators, namely social anxiety and mental well-being. Social anxiety emerges as a critical psychological response to excessive smartphone use, particularly through social media platforms that encourage comparison and validation seeking. This aligns with prior research highlighting how problematic smartphone use intensifies social anxiety and fear of negative evaluation [20, 21]. The elevated social anxiety then contributes to deteriorated mental well-being, reflected in emotional fatigue, isolation, and diminished self-esteem, consistent with findings from Abuhamdah and Naser [34] and Horwood and Anglim [93]. Finally, these deteriorations in mental well-being significantly undermine life satisfaction, echoing established evidence that mental health is a crucial determinant of overall life satisfaction [46, 48]. Therefore, the present study enriches the SPM by empirically demonstrating how modern digital behaviors, specifically smartphone addiction and social media use, fit within its framework of stress sources, mediators, and outcomes. It extends the model’s applicability to contemporary psychosocial stressors faced by university students in a technologically saturated environment.

### Significance and limitations

#### Theoretical contributions

This study offers three theoretical contributions. This research enhances the current literature on smartphone addiction, social anxiety for social media users, mental well-being and life satisfaction by proposing and empirically testing a serial mediation model that examines the impact of smartphone addiction on life satisfaction through the mediating effects of social anxiety for social media users and mental well-being among Chinese university students. This model builds upon previous research by integrating psychological factors (social anxiety among social media users and mental well-being) and behavioral factors (smartphone addiction) to elucidate the connection between smartphone addiction and life satisfaction.

Secondly, the study provides significant insights into the serial mediating effects of social anxiety for social media users and mental well-being, which have not been thoroughly investigated in prior studies. This study is among the first to incorporate social anxiety for social media users and mental well-being into a serial mediation framework, demonstrating how one mediates the impact of smartphone addiction on the other, subsequently influencing life satisfaction.

Thirdly, this study advances the theoretical development of the Stress Process Model [30] by applying it to the contemporary context of smartphone addiction among Chinese university students. According to the model, stress arises through three interrelated domains: sources of stress, mediators, and outcomes. In this study, smartphone addiction is conceptualized as the source of stress, which leads to increased social anxiety related to social media use and a decline in mental well-being, both functioning as mediators. These processes ultimately relate to lower life satisfaction, the manifestation of stress. By empirically demonstrating this serial mediation pathway, the study contributes an in-depth understanding of how digital-age stressors operate through interconnected psychological mechanisms. This finding builds upon and refines earlier research that examined mediators such as loneliness [22] or negative emotions [23] by introducing a more comprehensive sequential pathway grounded in a well-established theoretical framework. Moreover, it extends prior studies that employed parallel mediation [23] or single mediators [22] by offering a theoretically grounded, sequential explanation. This enriches the existing theoretical framework and provides a foundation for future research on technology-induced stress and subjective well-being.

#### Practical contributions

This study’s findings provide significant practical contributions that can enhance the mental well-being and life satisfaction of university students, especially those with smartphone addiction. These contributions can be utilized across multiple sectors, including higher education institutions, mental health services, and policy formulation. Universities are essential in fostering healthy habits, with physical activity serving as an effective means to improve life satisfaction, self-efficacy, and resilience among college students [94]. Promoting regular digital detoxes can significantly mitigate smartphone addiction. Universities could implement “unplugged” events or campaigns that require students to disconnect from their gadgets for designated intervals, facilitating face-to-face encounters, reducing social anxiety levels and enhancing mental well-being. These activities may also be associated with stress-reduction strategies, such as physical exercise or social bonding experiences, to enhance comprehensive student mental well-being and life satisfaction.

Educators and university personnel, particularly faculty counselors must be trained and educated to identify indicators of smartphone addiction, social anxiety, and inadequate mental well-being among students. By training teachers and counsellor staff with the ability to recognize these concerns promptly, the university can deliver appropriate interventions and support. This preventive strategy may mitigate the enduring impacts of smartphone addiction on students’ mental well-being and overall life satisfaction. Universities should initiate awareness efforts to emphasize the significance of life satisfaction and its correlation with digital behaviors. By instructing students about the effects of smartphone addiction on their mental well-being and overall contentment, institutions can enable them to make more educated choices regarding their digital usage. Such campaigns can also advocate for the advantages of a balanced, conscientious approach to technology utilization that increases life pleasure rather than diminishes it.

#### Limitations and future research

This study possesses several limitations that offer avenues for future investigation. The cross-sectional form of the research restricts the capacity to deduce causal links among smartphone addiction, social anxiety for social media users, mental well-being, and life satisfaction. This study’s design limits the ability to make strong causal inferences. It is possible that lower life satisfaction may contribute to higher smartphone addiction, indicating potential reverse causality. Future research should employ longitudinal designs to elucidate the causal dynamics over time and the enduring effects of smartphone addiction on students’ life satisfaction. Secondly, gender, age, education level, urban-rural residence, single-child versus multiple-child background, regional differences were incorporated as control variables. Additionally, several important control variables, such as personality traits, academic stress, and economic conditions, are recommended to be considered in future research, as their simultaneous effects on endogenous variable may introduce omitted variable bias.

Another limitation concerns the extremely high Cronbach’s alpha values, which exceed 0.95, particularly for the mental well-being scales. Nevertheless, the WEMWBS is a widely recognized and frequently used instrument for measuring individuals’ levels of positive mental well-being. While high internal consistency is generally desirable, values above 0.95 may suggest item redundancy. However, this study is consistent with prior research conducted in China by Chen et al. [95] and Lei et al. [96], where Cronbach’s alpha values similarly exceeded this threshold. Although common method bias was assessed and addressed in this study, future research is encouraged to critically evaluate the dimensionality of these scales and consider refining or shorter version of instrument to improve measurement precision and reduce potential redundancy.

In terms of sample representativeness, while the sample includes participants from all major regions of mainland China, the distribution is slightly skewed toward South, North, and Central China. Additionally, the gender distribution is imbalanced, with a higher proportion of female respondents, although this reflects the trend observed in some university populations in China [97]. The overrepresentation of gender and certain regions may limit the generalizability of the findings, hence future studies should aim for more balanced sampling to improve representativeness.

## Conclusion

This study provides a detailed examination of the complex relationship between smartphone addiction and life satisfaction among university students, focusing on the serial mediation effects of social anxiety for social media users and mental well-being. Guided by the Stress Process Model, the results offer valuable insights into the associations between smartphone addiction and lower life satisfaction, highlighting the potential indirect psychological pathways involving social anxiety related to social media use and mental well-being. The findings suggest that higher levels of smartphone addiction are associated with increased social anxiety for social media users and poorer mental well-being, which together relate to lower life satisfaction among Chinese university students. In other words, the association between smartphone addiction and life satisfaction occurs mainly through mediators such as social anxiety and mental health.

The study highlights the importance of university-based interventions that address smartphone addiction as a factor linked to life satisfaction. Interventions that aim to reduce social anxiety related to social media use and improve mental well-being may be important in efforts to enhance life satisfaction in this population. By exploring the psychological mechanisms that connect smartphone addiction to life satisfaction, this research contributes to a better understanding of how digital behaviors relate to the mental health of young adults and highlights the need for a holistic approach to student well-being.

Given the cross-sectional design, the findings do not establish causal relationships, and further longitudinal or experimental research is needed to clarify the directionality and causality of these associations. Additionally, future studies should explore how these relationships may vary across different cultural and educational contexts. Overall, this study adds to the growing body of literature on smartphone addiction and life satisfaction and provides a framework for future research and interventions aimed at supporting healthier and more satisfying university experiences in an increasingly digital world.

## Supplementary Information

Supplementary Material 1.

## Data Availability

The data supporting the findings of this research are obtainable upon request from the first author. Due to privacy or ethical restrictions, the data are not publicly accessible.

## Abbreviations

SAS: Smartphone addiction
SASSMUSCA: Social anxiety for social media users sharing content anxiety
SASSMUPCA: Social anxiety for social media users privacy concern anxiety
SASSMUIA: Social anxiety for social media users interaction anxiety
SSASSMUSEA: Social anxiety for social media users self-evaluation anxiety
MWB: Mental well-being
SWLS: Life satisfaction
SASSMU: Social anxiety for social media users
