# Supplementary material for: Smartphone addiction affects life satisfaction among Chinese university students: the serial mediation effects of social anxiety for social media users and mental well-being
Source: BMC Psychol. 2025 Oct 30;13:1200. doi: 10.1186/s40359-025-03544-9 (PMC12577056; doi:10.1186/s40359-025-03544-9)
Supplement: Supplementary file 1 — Supplementary Material 1. [file 40359_2025_3544_MOESM1_ESM.docx]

**Appendix A
Table A1. Measurement items of key variables**

| Variables | | Items | Source |
| --- | --- | --- | --- |
| **Smartphone Addiction Scale-Short Version (SAS-SV)** | **1** | Missing planned work due to smartphone use.  我曾因为使用手机而耽误计划好的学习或工作 | Kwon et al. (2013) [51]; Esmaeilpour et al. (2024) [52] |
|  | **2** | Having a hard time concentrating in class, while doing assignments, or while working due to smartphone use.  我曾因为使用手机在学习或工作时很难集中注意力 |  |
|  | **3** | Feeling pain in the wrists or at the back of the neck while using a smartphone.  使用手机时，我感到手腕或后颈部疼痛 |  |
|  | **4** | Won’t be able to stand not having a smartphone.  没有手机会使我难以忍受 |  |
|  | **5** | Feeling impatient and fretful when I am not holding my smartphone.  手机不在身边时，我会感到焦躁不安和不耐烦 |  |
|  | **6** | Having my smartphone in my mind even when I am not using it.  即使当我没有使用手机时，我也会一直想着它 |  |
|  | **7** | I will never give up using my smartphone even when my daily life is already greatly affected by it.  即使手机已经严重影响了我的生活，我也不会放弃使用手机 |  |
|  | **8** | Constantly checking my smartphone so as not to miss conversations between other people on Twitter or Facebook.  我经常查看手机，以免错过他人在社交网站上的留言 |  |
|  | **9** | Using my smartphone longer than I had intended.  我发现自己使用手机的时间比预期得长 |  |
|  | **10** | The people around me tell me that I use my smartphone too much.  我曾被告知在使用手机上花费太多时间 |  |
| **Social Anxiety for Social Media Users (SAS-SMU)** | **1** | I feel anxious about the fact that others might find my actions awkward.  我担心别人会觉得我的行为很尴尬 | Jia et al. (2022) [53] |
|  | **2** | I am concerned about being ridiculed by others for the content I have shared.  我担心自己分享的内容会被别人嘲笑 |  |
|  | **3** | I am concerned about the fact that the content I share will not be liked by others.  我担心我分享的内容别人不喜欢 |  |
|  | **4** | I am afraid that my close friends will not approve of my behavior.  我担心我的好朋友不支持我的行为 |  |
|  | **5** | I would feel uncomfortable when my friends publicly express their dislike about content I have shared.  当我的朋友公开表示不喜欢我分享的内容时，我会感到不舒服 |  |
|  | **6** | I am concerned about disapproval of my behaviors by others.  我担心别人不赞成我的行为 |  |
|  | **7** | I am concerned about being judged about my shared content by my friends in the presence of others.  我担心朋友在别人面前评判我分享的内容 |  |
|  | **8** | The possibility of having my private information acquired by others makes me feel anxious.  我的私密信息可能被别人获取让我感到焦虑 |  |
|  | **9** | The possibility of having my private information shared publicly makes me anxious.  我的私密信息可能被公开分享让我感到焦虑 |  |
|  | **10** | I feel uneasy when my friends share my private information with people I do not know.  当朋友与我不认识的人分享我的个人信息时，我会感到不安 |  |
|  | **11** | I would be concerned if my personal space is accessed without my consent.  当别人未经允许进入我的私人空间时，我会感到不安 |  |
|  | **12** | I feel anxious about how social media companies/ executives handle privacy policy regarding my private life.  我对社交媒体公司如何处理关于我私生活的隐私政策感到焦虑 |  |
|  | **13** | I feel anxious when talking with people I have just met.  和刚认识的人交谈时，我会感到焦虑 |  |
|  | **14** | I feel nervous when I talk with people I do not know very well.  和不太了解的人交谈时，我会紧张 |  |
|  | **15** | I feel uneasy while making new friends.  结交新朋友时，我会感到不安 |  |
|  | **16** | I feel tense when I meet someone for the first time.  和别人初次见面时，我会紧张 |  |
|  | **17** | I am afraid of interacting with others.  我害怕和别人交往 |  |
|  | **18** | I feel nervous when I have to talk with others about myself.  当我不得不和别人谈论自己时，我感到紧张 |  |
|  | **19** | I feel anxious about making a negative impression on people.  我担心我会给别人留下负面印象 |  |
|  | **20** | I am concerned about people thinking poorly of me.  我担心别人看不起我 |  |
|  | **21** | I feel anxious about not being able to meet people's expectations.  我对无法满足他人的期待而感到焦虑 |  |
| **Warwick-Edinburgh Mental Well-being Scale (WEMWBS)** | **1** | I've been feeling optimistic about the future.  面对未来我很乐观 | Tennant et al. (2007)[54] |
|  | **2** | I've been feeling useful.  我感觉自己很有用 |  |
|  | **3** | I've been feeling relaxed.  我感觉很放松 |  |
|  | **4** | I've been feeling interested in other people.  我很乐意和别人相处 |  |
|  | **5** | I've had energy to spare.  我感觉精力很充沛 |  |
|  | **6** | I've been dealing with problems well.  我能很好地处理问题 |  |
|  | **7** | I've been thinking clearly.  我的头脑一直都很清醒 |  |
|  | **8** | I've been feeling good about myself.  我一直都自我感觉良好 |  |
|  | **9** | I've been feeling close to other people.  我感觉和别人的关系很亲近 |  |
|  | **10** | I've been feeling confident.  我一直都很自信 |  |
|  | **11** | I've been able to make up my own mind about things.  对问题,我能做出自己的决定 |  |
|  | **12** | I've been feeling loved.  我一直都有被关爱的感觉 |  |
|  | **13** | I've been interested in new things.  我对新事物很感兴趣 |  |
|  | **14** | I've been feeling cheerful.  我感觉很愉快 |  |
| **Satisfaction with Life Scale (SWLS)** | **1** | In most ways my life is close to my ideal.  在多数情况下，我的生活接近我的理想 | Diener et al. (1985) [55]; Liu et al. (2024) [56] |
|  | **2** | The conditions of my life are excellent.  我的生活条件非常好 |  |
|  | **3** | I am satisfied with my life.  我满意自己的生活 |  |
|  | **4** | So far I have gotten the important things I want in life. 迄今为止，我已经得到生活中我想要的重要东西 |  |
|  | **5** | If I could live my life over, I would change almost nothing. 如果我可以重新生活，我基本不想改变什么 |  |
